# Rapamycin downregulates thymidylate synthase and potentiates the activity of pemetrexed in non-small cell lung cancer

**DOI:** 10.18632/oncotarget.1760

**Published:** 2014-02-16

**Authors:** Shigeru Kawabata, Chun-Te Chiang, Junji Tsurutani, Hideaki Shiga, Matthew L. Arwood, Takefumi Komiya, Joell J. Gills, Regan M. Memmott, Phillip A. Dennis

**Affiliations:** ^1^ Department of Oncology, Johns Hopkins Bayview Medical Center, Baltimore, MD, USA; ^2^ Medical Oncology Branch, Center for Cancer Research, National Cancer Institute, Bethesda, MD, USA; ^3^ Current address: Department of Otorhinolaryngology, Kanazawa Medical University, Ishikawa, Japan

**Keywords:** Rapamycin, Pemetrexed, Drug synergy, mTOR, Thymidylate Synthase, Lung Cancer

## Abstract

Non-small cell lung cancer (NSCLC) accounts for 80–85% of lung cancer cases, and almost half of newly diagnosed patients have metastatic disease. Pemetrexed is a widely used drug for NSCLC and inhibits several folate-dependent enzymes including thymidylate synthase (TS). Increased expression of TS confers resistance to pemetrexed *in vitro* and predicts poor response to pemetrexed. Rapamycin is an mTOR inhibitor and suppresses cap-dependent synthesis of specific mRNA species. Here, we show that the combination of rapamycin and pemetrexed synergistically inhibits proliferation of NSCLC cells. Although pemetrexed as a single agent induced TS, pretreatment with rapamycin suppressed pemetrexed-induced TS expression. *In vivo*, the combination of rapamycin and pemetrexed inhibited growth of NSCLC xenografts, which correlated with decreased mTOR activity and suppression of pemetrexed-induced TS expression. The ability of rapamycin to enhance the efficacy of pemetrexed and prevent TS expression has implications for the design of Phase I and/or Phase II NSCLC clinical trials with mTOR inhibitors in combination with pemetrexed.

## INTRODUCTION

Approximately 1.7 million new cancer cases and 0.6 million cancer deaths are projected to occur in the United States in 2013, and lung cancer is responsible for 26% and 28% of all female and male cancer deaths, respectively [1]. Non-small cell lung cancer (NSCLC) accounts for 80–85% of lung cancers, and 54% of patients with newly diagnosed NSCLC have advanced disease (i.e., distant stage or metastasis) [2]. Platinum-based chemotherapy regimens given as first-line treatment to advanced NSCLC patients with a good performance status have plateaued in overall response rate (25%-35%), median survival (8-10 mo.), one-year survival (30%-40%), and two-year survival (10%-15%) [3]. The best chemotherapy regimen for patients with advanced NSCLC remains to be determined.

Pemetrexed is a folate antimetabolite that is chemically similar to folic acid and inhibits three enzymes that contribute to purine and pyrimidine synthesis; thymidylate synthase (TS), hidydrofolate reductase (DHFR), and glycinamide ribonucleotide formyltransferase (GARFT) [4]. Pemetrexed was approved by the US Food and Drug Administration (FDA) as a first-line therapy for advanced non-squamous NSCLC patients when combined with cisplatin and as a maintenance therapy for patients with advanced non-squamous NSCLC who do not experience disease progression after platinum-based chemotherapy [5, 6]. Pemetrexed as a single-agent is a standard treatment for recurrent NSCLC patients who have previously received platinum-based chemotherapy, but the overall response rate is less than 10% and resistance to pemetrexed eventually develops [4]. The modest responses in the front line, second line, and maintenance settings provide rationale to develop new approaches to enhance the efficacy of pemetrexed.

Several mechanisms of resistance to antifolates have been described that include impaired influx, alteration of intracellular metabolism, increased target enzymes, an expanded folate pool, and increased efflux [7]. Increased expression of TS confers resistance to pemetrexed in lung cancer cells *in vitro* [8, 9] and is a predictive factor for poor response to pemetrexed in patients [10], indicating that inhibition of TS expression may be beneficial for overcoming resistance to pemetrexed.

The mammalian target of rapamycin (mTOR) is a serine/threonine protein kinase that belongs to the phosphoinositide 3-kinase (PI3K)-related kinase family, and controls protein synthesis, energy metabolism, cell proliferation, and survival [11]. Recently, it has been reported that pemetrexed can activate AMP-activated protein kinase (AMPK) through inhibition of a folate-dependent enzyme called aminoimidazolecarboxamide ribonucleotide formyltransferase (AICART), which indirectly inhibits mTOR and decreases phosphorylation of downstream substrates[12, 13], suggesting that mTOR inhibition can be a consequence of anti-folate activities.

Previously we reported that rapamycin, an mTOR inhibitor, prevented the development of tobacco carcinogen–induced lung tumors in a mouse model, suggesting that the mTOR pathway is implicated in lung tumorigenesis [14]. Because rapamycin has broad anti-proliferative activity across a series of NSCLC cell lines, we hypothesized that combining rapamycin with pemetrexed might enhance mTOR inhibition, suppress TS expression, and synergistically decrease proliferation of NSCLC cells. Our data confirm that rapamycin enhances the efficacy of pemetrexed and suppresses pemetrexed-induced TS expression *in vitro* and *in vivo*.

## RESULTS

**Figure 1:**
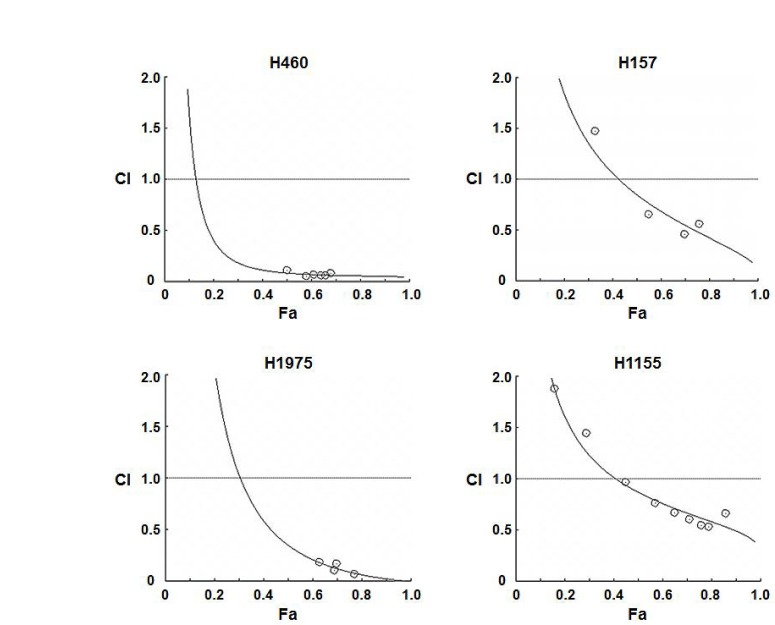
Pemetrexed and rapamycin synergistically inhibit the proliferation of NSCLC cells *in vitro* NSCLC cells were treated with 0.1% DMSO or varying concentrations of pemetrexed, rapamycin, or the combination for cell proliferation assays and assessment of CI values as described in Materials and Methods. Computer-simulated Fa-CI curves show synergism (CI < 1), additive effect (CI = 1), or antagonism (CI > 1) for the indicated levels of growth inhibition (Fa) induced by the drug combination (see also Supplementary Table 1A). Circles (O) indicate the Fa-CI data points based on experimental values (see also Supplementary Table 1B).

To assess effects on cellular proliferation, rapamycin and pemetrexed were tested at concentrations that are clinically achievable in a series of four NSCLC cell lines that vary in status of molecular targets such as EGFR and Kras. The inhibitory concentration 50 (IC50) values of each drug when used in combination were less than when used as single treatments, indicating synergistic effects at the effective dose 50 (ED50) (Table 1). Computer-simulated Fa-CI curves showed synergism (CIs <1) in the four cell lines evaluated (Figure 1 and Supplementary Table 1). These data suggest that rapamycin enhances the anti-proliferative effects of pemetrexed.

**Table 1:** IC50 values of pemetrexed and rapamycin in either single or the combination treatment

		Single treatment			Combination treatment		
Cell line	ExposureTime	Pemetrexed	Rapamycin		Pemetrexed	Rapamycin	CI at ED50
	(hr)	(nM)	(nM)		(nM)	(nM)	
H460	72	308.96	41.27	19.28	0.96	0.09
H157	96	77.16	2.51	58.36	0.23	0.85
H1975	96	581.56	4.03	52.23	1.04	0.35
H1155	72	73.03	1.43	42.06	0.42	0.87

NSCLC cells were treated with 0.1% DMSO as a control or varying concentrations of pemetrexed, rapamycin, or the combination for cell proliferation assay and assessment of CI values as described in Materials and Methods. Inhibitory concentration 50 (IC50) is the concentration of an inhibitor where the response is reduced by half, whereas effective dose 50 (ED50) is the concentration of a drug that gives half-maximal response. Combination Index (CI) values < 1 indicate synergism. See also Supplementary Table 1.

**Figure 2:**
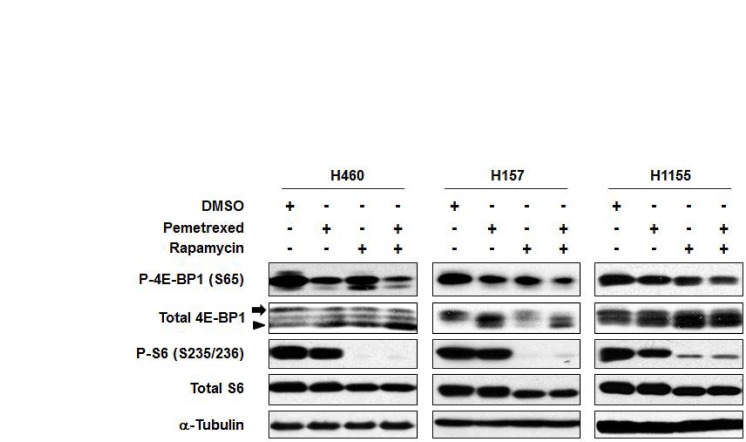
The combination of rapamycin and pemetrexed inhibits the mTOR pathway to a greater extent than either drug alone in a panel of NSCLC cell lines IC50 concentrations were used for these studies. Specifically, H460 cells were treated with either 309 nM pemetrexed, 41 nM rapamycin, or the combination for 72 hr; H157 cells were treated with either 77 nM pemetrexed, 2.5 nM rapamycin, or the combination for 96 hr; and H1155 cells were treated with either 73 nM pemetrexed, 1.4 nM rapamycin, or the combination for 72 hr (see also Table 1). As a control, the indicated cell lines were treated with 0.1% DMSO. Immunoblotting analysis was performed for components of the mTOR pathway. The arrow and arrowhead indicate hyper- and hypophosphorylated forms of 4E-BP1, respectively.

Because rapamycin is a prototypic mTOR inhibitor and pemetrexed has been reported to decrease mTOR activity in a small number of cell lines, mTOR inhibition was assessed in a series of NSCLC cell lines. Phosphorylation of the ribosomal protein S6 and 4E-BP1 was measured as a surrogate for mTOR activity. S6 is phosphorylated by S6K, which is a direct substrate of mTOR. 4E-binding protein 1 (4E-BP1) is regulated by mTOR and inhibits 5′-cap-dependent mRNA translation by binding and inactivating eukaryotic translation initiation factor 4E (eIF4E) that is involved in the mRNA-ribosome binding step of eukaryotic protein synthesis. Phosphorylated 4E-BP1 by mTOR is dissociated from eIF4E, resulting in increasing protein synthesis [11]. As a single agent, pemetrexed decreased phosphorylation of S6 and 4E-BP1 in H460, H157 and H1155 cells, but to a lesser extent than rapamycin as a single agent. Combining rapamycin with pemetrexed decreased phosphorylation of 4E-BP1 and increased hypophosphorylated forms of 4E-BP1, but the effects of the combination on phosphorylation of S6 were less apparent because rapamycin was highly effective at inhibiting S6 phosphorylation as a single agent (Figure 2). These results suggested that >the combination of rapamycin and pemetrexed enhances the inhibition of the mTOR pathway, but also raised the possibility that other molecular targets might be responsible for the combined anti-proliferative effects.

**Figure 3:**
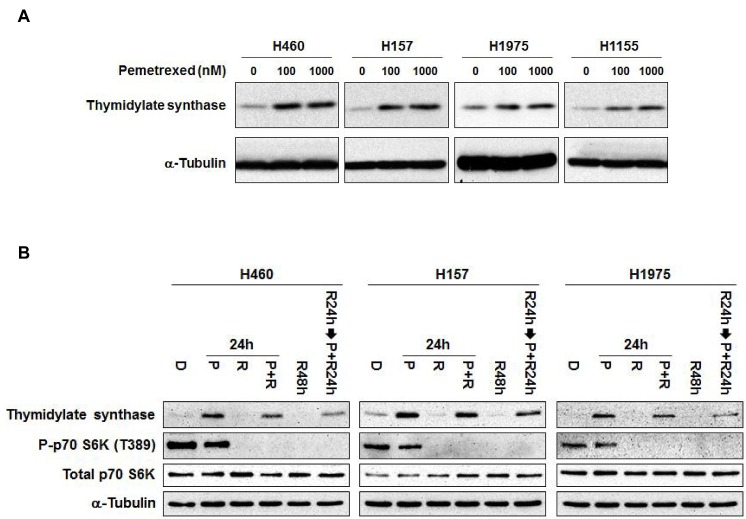
Rapamycin prevents pemetrexed-induced increases in TS expression (A) Pemetrexed induces the expression of TS. NSCLC cells were treated with pemetrexed at the indicated concentrations for 24 hr. Levels of TS expression were assessed by immunoblotting analysis. (B) Pretreatment of cells with rapamycin improves its ability to suppress pemetrexed-induced increases in TS expression. D: 0.1% DMSO for 48 hr; P: 500 nM pemetrexed for 24 hr; R: 100 nM rapamycin for 24 hr; R48h: 100 nM rapamycin for 48 hr; R24h→P+R24h: pretreatment with 100 nM rapamycin for 24 hr followed by combining 500 nM pemetrexed with 100 nM rapamycin for 24 hr. Immunoblotting analysis was performed for the indicated biomarkers.

An increased basal level of TS expression is recognized as a mechanism of resistance to pemetrexed [8, 9]. Pemetrexed has been reported to inhibit TS activity in NSCLC patients [15], but high concentrations of pemetrexed can induce TS expression in A549 cells [16]. To clarify the effects of pemetrexed on TS expression, two concentrations of pemetrexed were used in four NSCLC cell lines (Figure 3A). Our results show that pemetrexed potently induced TS expression, because 100 nM increased TS expression in all four cell lines. To assess the effects of combining rapamycin and pemetrexed, simultaneous administration for 24 hr and sequential administration (rapamycin for 24 hr followed by the addition of pemetrexed for 24 hr) were compared (Figure 3B). As a single agent given for 24 hr, pemetrexed induced TS expression and modestly decreased phosphorylation of S6K in the three cell lines tested. Rapamycin as a single agent markedly decreased S6K phosphorylation in all cell lines tested, and modestly decreased the basal level of TS in H157 cells. When rapamycin and pemetrexed were administered simultaneously, rapamycin only modestly decreased the expression of TS that was induced by pemetrexed, suggesting that pretreatment with rapamycin might prevent pemetrexed-induced TS expression to a greater extent. Indeed, pretreatment of cells with rapamycin substantially improved its ability to inhibit pemetrexed-induced increases in TS expression. Collectively, these findings suggest that rapamycin might increase the efficacy of pemetrexed by preventing pemetrexed-induced TS expression.

**Figure 4:**
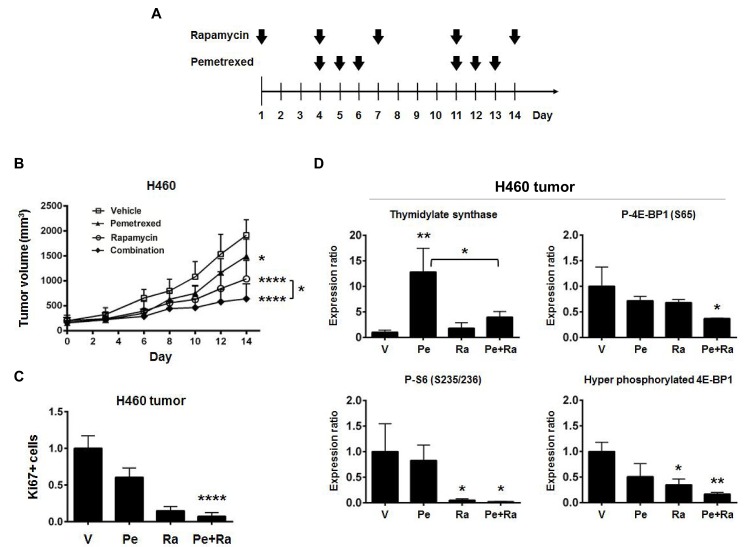

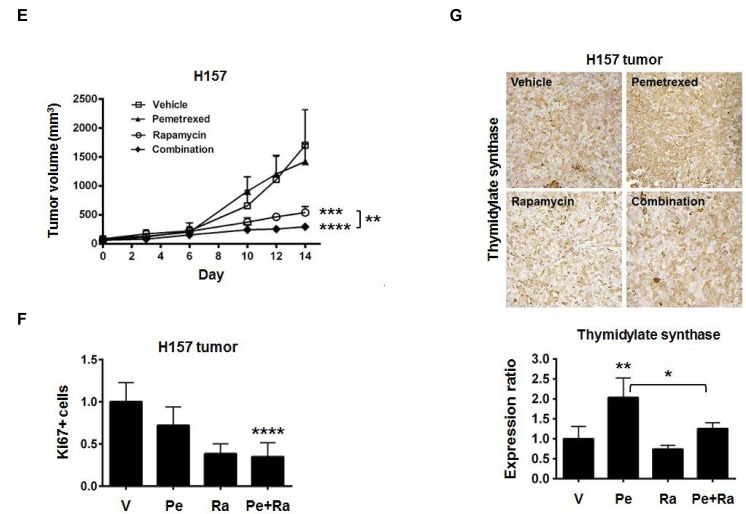
Combining rapamycin with pemetrexed inhibits the growth of NSCLC xenograft tumors to a greater extent than either drug alone (A) The schema for NSCLC xenograft tumor treatments *in vivo* shows a lead in period of rapamycin alone followed by treatment with both rapamycin and pemetrexed. (B) H460 cells were grown as xenografts in athymic NCr-nu/nu. bars, SD. *, p <0.05; ****, p <0.0001 for vehicle treatment. (C) Inhibition of cell proliferation in H460 tumors was assessed by immunohistochemistry for Ki67. The staining index was determined as described in Materials and Methods, and then normalized to vehicle for each treatment. Columns, mean from three of five mice examined in H460 xenografts. bars, SD. ****, p <0.0001 for vehicle treatment. (D) Biomarker analysis for the combination of rapamycin and pemetrexed *in vivo*. At the end of the study, H460 xenograft tumors were excised and processed for immunoblotting analysis of the indicated markers as described in Materials and Methods. Densitometry was performed using ImageJ version 1.43 software (http://rsbweb.nih.gov/ij/index.html). The levels of each marker were normalized to α-Tubulin for each sample. Columns, show mean values from 3 mice. bars, SD. *, p <0.05; **, p <0.01 for vehicle treatment. (E) H157 cells were grown as xenografts in athymic NCr-nu/nu. bars, SD. **, p <0.01 (unpaired t test); ***, p <0.001; ****, p <0.0001 for vehicle treatment. (F) Inhibition of cell proliferation in H157 tumors. Ki67+ cells were assessed by immunohistochemistry. The staining index was determined as described in Materials and Methods, and then normalized to vehicle for each treatment. Columns, mean from three of five mice examined in H157 xenografts. bars, SD. ****, p <0.0001 for vehicle treatment. (G) Rapamycin suppresses pemetrexed-induced increases in TS expression *in vivo*. Levels of TS were assessed by immunohistochemistry of H157 xenograft tumors excised at the end of the study. The TS staining index was determined as described in Materials and Methods, and then normalized to vehicle for each treatment. Columns, mean from all 5 mice examined in H157 xenografts. bars, SD. *, p <0.05; **, p <0.01 for vehicle treatment.

To determine if the combination of rapamycin and pemetrexed has enhanced anti-tumor effects, athymic NCr-nu/nu mice bearing established H460 (Figure 4B-D) or H157 (Figure 4E-G) tumor xenografts were treated with either vehicle, 2.5 mg/kg rapamycin, 100 mg/kg pemetrexed, or the combination (Figure 4A). The treatment schedule was designed to allow for a three day window of rapamycin pretreatment prior to simultaneous treatment. The combination was well tolerated and decreased H460 tumor growth by nearly 70%, which was greater than inhibition due to either drug alone (*p* <0.0001, Figure 4B). The staining for the nuclear-specific antigen Ki67 revealed a significantly lower rate of proliferation in combination-treated H460 tumors (*p* <0.0001, Figure 4C). Pemetrexed-induced TS expression in tumors was suppressed by rapamycin (*p* <0.05, Figure 4D), and 4E-BP1 phosphorylation was decreased by the combination. A further decrease in phosphorylation of S6 was not observed in tumor tissues, which is likely the result of effective inhibition of S6 phosphorylation by rapamycin alone. In H157 tumors, the combination of rapamycin and pemetrexed was also effective, decreasing growth by nearly 80% (*p* <0.0001, Figure 4E) and inhibiting cellular proliferation (*p* <0.0001, Figure 4F). As observed in H460 tumors, rapamycin also suppressed pemetrexed-induced TS expression as assessed by immunohistochemistry (*p* <0.05, Figure 4G) (immunoblotting was not possible due to fixation of the tumors). These data suggest that rapamycin improves the inhibition of NSCLC tumor growth by preventing pemetrexed-induced expression of TS.

## DISCUSSION

We investigated new approaches to enhance the efficacy of pemetrexed, and found that the combination of rapamycin and pemetrexed showed synergistic anti-proliferative effects in NSCLC cells and enhanced mTOR inhibition, as demonstrated by decreases in the phosphorylation of the downstream components of this pathway such as 4E-BP1. Furthermore, rapamycin prevented pemetrexed-induced TS expression *in vitro* and *in vivo*. To our knowledge, this is the first study to describe the benefit of using an mTOR inhibitor to suppress the induction of TS that occurs as a compensatory response to pemetrexed in lung cancer model systems. Similar results have been reported in gastric cancer cell lines. Shigematsu, *et al*. reported that rapamycin decreased the endogenous expression levels of TS in gastric cancer cell lines, which enhanced chemotherapy-induced cytotoxicity [17]. Additionally, RAD001, a derivative of rapamycin, also decreased endogenous TS protein levels in gastric cancer cell lines. The combination of RAD001 with the anti-metabolite 5-fluorouracil significantly decreased TS protein levels to a greater extent than with RAD001 alone [18]. Collectively, these studies suggest that in general, mTOR inhibition is an effective way to decrease TS expression and increase the response to pemetrexed.

Despite the efficacy of the combination in the NSCLC cell lines we tested, our results are in conflict with those of Markova *et al*. [19], who showed that RAD001 protected NSCLC cells from pemetrexed-induced apoptosis. They concluded that mTOR inhibitors might suppress the antitumor activity of pemetrexed by slowing cell cycle progression. What might explain this discrepancy?In our study, the combination of rapamycin and pemetrexed showed synergistic effects in H460 and H1975 cells that have PIK3CA (phosphatidylinositol-4,5-bisphosphate 3-kinase, catalytic subunit alpha) mutations, and in H157 and H1155 cells that have PTEN (phosphatase and tensin homolog) mutations. Each of these mutations leads to increased mTOR activity. In contrast, the NSCLC cell lines used by Markova *et al*. do not have PIK3CA or PTEN mutations. To further evaluate PIK3CA or PTEN mutations as predictive for response to the combination, we evaluated the effects of combining rapamycin with pemetrexed with other human NSCLC cell lines that do not have PIK3CA or PTEN mutations (H1703 and PC-9 (formerly known as PC-14) cells). Rapamycin did not enhance the efficacy of pemetrexed in these cell lines (data not shown). This is consistent with the results of Meric-Bernstam*et al*., who reported that human cancer cells with PIK3CA and/or PTEN mutations were more likely to be sensitive to rapamycin [20]. Taken together, these findings suggest that PIK3CA or PTEN mutations contribute to the synergistic effects of combining rapamycin with pemetrexed.

Several reports show that pemetrexed-resistant lung cancer cell lines treated for 2 to 6 months have significantly increased levels of TS protein compared to the parental cell lines [8, 21]. On the other hand, we found that short-term pemetrexed treatment induced TS expression, which was suppressed by rapamycin. Pretreatment of cells with rapamycin prior to administration of pemetrexed enhanced the suppression of pemetrexed-induced TS expression compared to the combination without rapamycin-pretreatment (Figure 3B). What mechanisms might underlie inhibition of TS by rapamycin? Because rapamycin inhibits protein synthesis, it is likely to suppress the translation of ribosome-associated TS transcripts that are induced by pemetrexed. In addition, Lee *et al*. reported that the mechanism of TS protein down-regulation by RAD001 might be dependent on proteasomal degradation, because the treatment of a proteasome inhibitor blocked the down-regulation of TS protein by RAD001 [18]. Future studies might clarify the roles of the each of these mechanisms and/or identify additional mechanisms of TS suppression by mTOR inhibition.

The results of these studies have clinical implications. TS may be a valuable target in NSCLC. For example, Kaira *et al*. reported that positive TS expression in tumor tissues from 160 patients with completely resected NSCLC was significantly associated with advanced stage and lymph node metastases, and was an independent prognostic factor for predicting a poor outcome in patients with lung adenocarcinoma [22]. A recent meta-analysis that evaluated the predictive value of TS in pemetrexed-containing chemotherapy regimens for NSCLC patients suggests that increased levels of TS are an independent risk factor for potential resistance against pemetrexed [10]. Early clinical trials combining mTOR inhibitors with pemetrexed have been completed. A phase I study of RAD001 given simultaneously with pemetrexed in previously treated NSCLC patients has been reported, and the regimen was well tolerated with a 42% disease control rate (partial response (PR) and stable disease) [23]. These results are similar to what might be expected for pemetrexed alone, but biomarkers and TS levels were not analyzed in this study. A hypothetical advantage of using rapamycin includes the fact that rapamycin can protect normal cells from the cytotoxicity of chemotherapeutic agents and radiation-induced damage, but sensitize cancer cells to these therapeutic modalities [24, 25]. We recently completed a Phase I/II clinical trial combining rapamycin and pemetrexed in patients with relapsed NSCLC who had any number of prior treatments, and found that the regimen was well tolerated with a 22% PR rate (manuscript in preparation). We incorporated a one-week lead in period with rapamycin alone for pharmacokinetics and biomarker analysis. Immunoblotting analysis of peripheral blood mononuclear cells collected from patients showed that rapamycin alone decreased the level of endogenous expression of TS, which correlated with progression free survival and was consistent with our preclinical results [26]. These results suggest that the maximum clinical benefit of combining mTOR inhibition with pemetrexed might be achieved through a lead in period with mTOR inhibition alone. In addition, future studies should assess basal levels of TS expression in tumor tissue and stratify patients based on status of PIK3CA and PTEN to validate potential predictive markers.

## MATERIALS AND METHODS

### Cell culture

NSCLC (H460, H157, and H1155) cell lines with Kras mutations were established at the National Cancer Institute (NCI, Bethesda, MD, USA), and the NSCLC H1975 cell line with EGFR mutations was a kind gift from Dr. William Pao (Vanderbilt-Ingram Cancer Center, Nashville, TN, USA). All cell lines were maintained in 75 cm2 flask in RPMI 1640 (Life Technologies, Grand Island, NY, USA) with 5% fetal bovine serum (FBS, Life Technologies) at 37○C in a 5.0% CO2 atmosphere incubator. LKB1 mutations are detected in H460 and H157 cell lines. The mutation status of NSCLC cells was obtained from Cell Line Project, Catalog of Somatic Mutations in Cancer (COSMIC), the Wellcome Trust Sanger Institute Cancer Genome Project website (http://cancer.sanger.ac.uk/cancergenome/projects/cosmic/).

### Reagents

Rapamycin and pemetrexed were obtained from LC Laboratories (Woburn, MA, USA). Primary antibodies for P-4E-BP1 (Ser65), 4E-BP1, P-S6 Ribosomal Protein (Ser235/236), S6 Ribosomal Protein, thymidylate synthase (TS), P-p70 S6 Kinase (Thr389), and p70 S6 Kinase were from Cell Signaling Technology (Beverly, MA, USA). Anti-α-Tubulin and Ki67 antibodies were from Sigma (St. Louis, MO, USA) and Abcam (Cambridge, MA, USA), respectively.

### Cell proliferation assay and Combination Index (CI)

NSCLC cells (2,500 cells per well) were plated in 96-well plates and allowed to grow overnight. Cells were treated with rapamycin dissolved in DMSO, pemetrexed dissolved in PBS, or the combination in a wide concentration range between 0.01 nM and 10000 nM for 72 hr (H460 and H1155 cells) or 96 hr (H157 and H1975 cells). Growth inhibition was determined by the sulforhodamine B assay [27]. Percent growth value was calculated by using the absorbance values of untreated cells on day 0 (D0), DMSO-treated control cells (C), and drug-treated cells (T) as follows: [(T - D0)/(C - D0)] x 100 for concentrations for which T>/=D0, or [(T - D0)/C] x 100 for concentrations for which T<D0. Experiments were performed three times, and each drug concentration was evaluated in sextuplet wells for a given experiment. CI is a quantitative measure of the degree of drug interaction in terms of synergism (CI < 1), additive effect (CI = 1), or antagonism (CI > 1) for a given endpoint of the effect measurement [28]. Additive effect is defined as the combined effect predicted by the mass-action law principle, synergism is as the production of a greater than expected additive effect, and antagonism is as the production of smaller than expected additive effect. Inhibitory concentration 50 (IC50) and Combination Indices (CIs) were calculated using CompuSyn software program (ComboSyn, Inc., Paramus, NJ, USA). Affected fraction (Fa) is defined as a function of effect level (e.g., degree of inhibition) by a dose of drug. Fa values were calculated according to the program's instruction as follows: [(100 - %1"> growth value)/100], which indicated a growth inhibition value.

### Immunoblotting

Cells (5 x 10^5^ cells per well) were plated in six-well plates. The following day, cells were treated with drug or equal volume of DMSO for the indicated times and lysed in 2 x lysis buffer as described previously [29]. For tumor-tissue homogenates *in vivo*, frozen tumors were allowed to thaw on ice, then homogenized in radioimmunoprecipitation assay buffer [150 mmol/L NaCl, 1% Igepal CA-630, 0.5% sodium deoxycholate, 0.1% SDS, 50 mmol/L Tris (pH 8.0)] containing 2.5 mol/L h-glycerol phosphate, 0.2 mol/L sodium orthovanadate, 1.25 mol/L sodium fluoride, and 1 x protease inhibitor cocktail (Roche Diagnostics, Indianapolis, IN, USA) using a hand-held Tissue-Tearor homogenizer (Biospec Products, Bartlesville, OK, USA). Cell lysates or tumor-tissue homogenates with equal amounts of protein were separated by SDS-PAGE then transferred to nitrocellulose membranes. The membranes were blocked for 1 hr in blocking buffer (1 x TBS, 5% milk, 0.1% Tween 20) and placed in primary antibody diluted in 1 x TBS, 5% bovine serum albumin, 0.1% Tween 20, overnight at 4°C. The following day, membranes were washed thrice in wash buffer (0.1% Tween 20, 1 x TBS). Primary antibody was detected using horseradish peroxidase–linked secondary antibodies and visualized with the enhanced chemiluminescent detection system (Amersham Biosciences, Pittsburgh, PA, USA). Immunoblot experiments were performed at least 3 times.

### Drug treatment in vivo

Six-week-old female athymic NCr-nu/nu mice (Charles River Labs, Frederick, MD, USA) were injected subcutaneously (s.c.) in both rear flanks with 5 x 10^6^ H460 or H157 cells in 100 μl PBS. When the transplanted tumors reached a volume of 50 mm^3^, mice were divided into the following four groups (5 mice per group): intraperitoneal (i.p.) injection of either (1) vehicle (4% DMSO, 5% PEG, 5% Tween 80 in saline; once daily on days 1, 4 to 7, and 11 to 14), (2) >2.5 mg/kg rapamycin (once daily on days 1, 4, 7, 11, and 14) as described previously [14], (3) 100 mg/kg pemetrexed (once daily on days 4 to 6 and 11 to 13) based on tolerated dosage as described previously [30], or (4) the combination of rapamycin and pemetrexed (once daily on each drug's schedule). Animal weights and tumor measurements were made every other day. In all studies, tumor volume was calculated from the formula v = (*ab*^2^) / 2, where *a* is the long axis and *b* is the short axis. *In vivo* experiments were conducted under a protocol approved by the NCI Animal Care and Use Committee.

### Immunohistochemistry

Formalin-fixed, paraffin-embedded xenograft tumor tissues were sectioned, placed on poly-L-lysine–coated slides (Histoserv Inc., Germantown, MD, USA), and analyzed for protein expression for five mice per group. Antigen retrieval was carried out using preheated target retrieval solution (pH 6.0) from DakoCytomation (Carpinteria, CA, USA) for 30 min in a boiling rice cooker. Vectastain Elite ABC kits from Vector Laboratories (Burlingame, CA, USA) were used according to manufacturer's instructions for blocking, dilution of primary antibody, and labeling. Primary antibody was incubated with sections for 16 hr at 4°C. 3,3- Diaminobenzidine was prepared fresh from tablets (Sigma). Specificity of staining was assessed by comparison with samples stained in the absence of primary antibody. All slides were blinded to the investigators before scoring, and in all cases, xenograft tumors were assessed for three to five mice per group. The staining index of TS was determined by assigning a score of absent (0), minimal (1), moderate (2), or high (3) staining to each cell in five 400-highpower fields (HPF). The staining index was then calculated by multiplying the staining intensity by its distribution scored as 0 (0%), 1 (1% to 20%), 2 (21% to 40%), 3 (41% to 60%), 4 (61% to 80%), 5 (81% to 100%) to indicate the percentage of positive cells of interest in a single core. Ki67 staining was quantified by counting the number of the positive cells in five 400-HPF per tumor. Numbers were averaged for three to five mice per group. An overall score was assigned to each slide, and the scores were averaged for vehicle versus drug-treated groups.

### Statistics analysis

Statistical significance of differences observed in drug-treated and untreated cells was analyzed using one-way/two-way ANOVA, and then multiple comparisons were performed by Bonferroni test. All analyses were performed using the GraphPad Prism software version 5.0c. The threshold value was set to 0.05.

## SUPPLEMENTARY TABLES


