# Supplementary material for: Rapamycin downregulates thymidylate synthase and potentiates the activity of pemetrexed in non-small cell lung cancer
Source: Oncotarget. 2014 Feb 16;5(4):1062–70. doi: 10.18632/oncotarget.1760 (PMC4011583; doi:10.18632/oncotarget.1760)
Supplement: Supplementary file 1 [file oncotarget-05-1062-s001.pdf]

Rapamycin downregulates thymidylate synthase and potentiates the activity of pemetrexed in non-small cell lung cancer – Kawabata et al

Supplementary Table 1A

CI simulations				
	H460	H157	H1975	H1155
Fa	CI	CI	CI	CI
0.10	1.66	2.95	5.95	2.56
0.20	0.40	1.84	2.06	1.60
0.30	0.19	1.35	1.03	1.23
0.40	0.12	1.06	0.58	1.02
0.50	0.09	0.85	0.35	0.87
0.60	0.07	0.69	0.21	0.76
0.70	0.06	0.55	0.12	0.67
0.80	0.06	0.42	0.06	0.58
0.90	0.05	0.30	0.02	0.49
0.97	0.05	0.20	0.01	0.40

Supplementary Table 1B

CIs for experimental values							
H460		H157		H1975		H1155	
Fa	CI	Fa	CI	Fa	CI	Fa	CI
						0.16	1.88
						0.29	1.45
						0.45	0.97
0.50	0.11					0.57	0.76
0.58	0.06					0.65	0.67
0.61	0.07	0.33	1.47	0.63	0.18	0.71	0.61
0.66	0.06	0.55	0.65	0.69	0.11	0.76	0.55
0.68	0.08	0.70	0.46	0.70	0.17	0.79	0.53
0.64	0.06	0.76	0.56	0.77	0.07	0.86	0.66

Supplementary Table 1: Combination Index (CI) values in the combination of pemetrexed and rapamycin. NSCLC cells were treated with 0.1% DMSO as a control or varying concentrations of pemetrexed, rapamycin, or the combination for cell proliferation assays and assessment of CI values as described in Materials and Methods. CI simulations display synergism (CI < 1), additive effect (CI = 1), or antagonism (CI > 1) for the entire spectrum of effect levels (Fa) in the combination of pemetrexed and rapamycin **(A)**. CIs for experimental values **(B)** are displayed as actual Fa-CI plots in Figure 1. The gray highlight indicates synergism.
